# The cost-effectiveness of placement restrictions of unhealthy food and drinks at end of aisles and checkouts in Australian supermarkets

**DOI:** 10.1186/s12937-026-01307-9

**Published:** 2026-03-13

**Authors:** Bisola Osifowora, Huong Tran, Emma Frew, Jaithri Ananthapavan

**Affiliations:** 1 Centre for Economics of Obesity, University of Birmingham, Birmingham, B15 2TT UK; 2 Faculty of Health, School of Health and Social Development, Institute for Health Transformation, Deakin Health Economics (DHE) Geelong, Deakin University, VIC, Australia; 3 Faculty of Health, School of Health and Social Development, Institute for Health Transformation, Global Centre for Preventive Health and Nutrition (GLOBE), Deakin University, Geelong, VIC Australia

**Keywords:** ACE-Obesity Policy model, Cost-effectiveness, Health policy, Obesity prevention, Retail marketing restrictions, Supermarkets.

## Abstract

**Background:**

Obesity is a significant contributor to global morbidity and mortality, driven by rising consumption of unhealthy energy dense foods high in salt, sugar and fat (HFSS). Supermarkets, pivotal in food distribution, influence consumer choices through marketing strategies that regularly promote the purchase of unhealthy foods. Restricting the placement of unhealthy food and beverages in prominent supermarket locations is a promising intervention to reduce the purchase of unhealthy products. This study assesses the cost-effectiveness of a potential policy to restrict the placement of unhealthy foods at prominent locations in supermarkets like checkouts, end-of-aisles, and store entrances compared to the status quo (no policy).

**Methods:**

The impact of the proposed policy to restrict the placement of unhealthy foods on the distribution of Body Mass Index (BMI) in the 2024 Australian population was estimated. A previously validated multiple cohort Markov model (Assessing Cost Effectiveness-Obesity Policy model) was used to estimate the impact of the change in BMI on the epidemiology of nine obesity related diseases resulting in changes in long-term health outcomes quantified as health-adjusted life years (HALYs), and healthcare costs. Policy implementation and monitoring costs to government and retailers were estimated. The analyses were undertaken over the lifetime of the modelled population (100 years), adopting a limited societal perspective with results presented in 2024 values. Deterministic and probabilistic sensitivity analyses were performed to evaluate the robustness of the results.

**Results:**

The placement restriction policy was estimated to result in a mean BMI reduction of 0.26 kg/m² for males and 0.21 kg/m² for females, corresponding to 0.37 million HALYs gained and reduced healthcare costs of A$4.45 billion over the lifetime of the population. The total cost of the policy was A$20 million. Sensitivity analyses confirmed the intervention’s cost-effectiveness across various scenarios.

**Conclusion:**

A policy to restrict retail marketing of unhealthy foods in supermarkets represents a potentially cost-effective intervention, estimated to produce long-term health gains and save costs. These findings support policy action to create healthier food retail environments.

**Supplementary Information:**

The online version contains supplementary material available at 10.1186/s12937-026-01307-9.

## Background

Obesity is a significant contributor to global illness and death, primarily due to its role as a major risk factor for several non-communicable diseases (NCDs) [1–4]. Despite widespread recognition of this problem, no country has successfully reversed the rising prevalence of overweight and obesity, as systemic, environmental and institutional factors driving obesity remain largely under addressed [5].

Australia is among the countries most affected by overweight and obesity, with approximately 31% of adults affected, reflecting one of the highest rates worldwide [6]. In addition to health impacts, the financial implications of obesity are substantial, impacting individuals, communities, and national economies [6]. In Australia, the 2022–2032 National Obesity Strategy estimates that without intervention, obesity will cost approximately A$11.8 billion annually [6]. Australians spend 58% of their average household food budget on unhealthy foods and beverages, which accounts for 35% of the daily energy intake for both adults and children [6, 7].

Research has highlighted the significant role of the food system in shaping these dietary behaviours [5, 8, 9]. Supermarkets play a central role in food distribution, particularly in high-income countries, and are increasingly becoming the norm in low- and middle-income countries [4, 8]. Their accessibility, through convenient locations, extended operating hours, and wide range of product offerings makes them the primary food source for many households [3]. Marketing studies have shown that two-thirds of in-store food purchasing decisions are unplanned, demonstrating the impact of in-store marketing techniques on consumer purchasing behaviour [3]. Food manufacturers often pay for prominent shelf space or promotional displays to maximise visibility for their products to enhance product sales [10].

Australia’s National Obesity Strategy has proposed actions involving working with supermarket chains to stock healthier, affordable options aligned with the Australian Dietary Guidelines (ADG), while reducing the placement of unhealthy products in prominent locations [6, 11]. This approach aims to create supportive environments that empower consumers to make healthier choices [6]. Several studies have assessed the effectiveness of restricting unhealthy food and beverage placements in supermarkets on purchasing behaviours. These studies consistently show that such interventions reduce sales of unhealthy products [2, 12–18]. In addition to the effectiveness of polices, evidence from economic evaluations can provide valuable insights on the economic value of policy options to guide government resource allocation decision-making [1, 2, 7]. This study was designed to evaluate the cost-effectiveness of restricting the placement of unhealthy foods in prominent supermarket locations in Australia.

## Methods

A cost-effectiveness evaluation was conducted using a cost-utility analysis framework with health-adjusted life years (HALYs) as the outcome measure, to assess the value for money of a potential policy to restrict the placement of unhealthy foods in prominent locations within major Australian supermarkets from a limited societal perspective. The primary analysis assessed a lifetime horizon (100 years) to estimate the costs of implementation and the benefits in terms of health and reduced healthcare costs for the 2024 Australian population. Costs and benefits were discounted at 3% [19] and presented in 2024 values. The analysis involved four key stages. In the first stage, a logic pathway was developed to estimate the costs of intervention implementation and its impact on population body mass index (BMI). The second stage translated population changes in BMI to changes in long-term health outcomes and healthcare-related costs using the Assessing Cost-Effectiveness (ACE)-Obesity Policy model [20]. The third stage aggregated the incremental costs and health outcomes of the intervention relative to the comparator, to calculate the incremental cost-effectiveness ratio (ICER). The final stage involved conducting both scenario (Table 1) and probabilistic sensitivity analysis (PSA) to assess the robustness of the results. All results are presented as mean values with 95% uncertainty intervals (UIs).

### Intervention

The intervention was defined as government legislation to restrict the placement of unhealthy foods in prominent locations within major Australian supermarkets (Woolworths, Coles, Aldi, and IGA, who account for 82.4% of the Australian supermarket market share) [10, 21]. The ADG recommends limiting the consumption of unhealthy foods, which are defined as energy-dense, nutrient-poor products. In this analysis, unhealthy foods were classified using a category-based approach and included chocolates, confectionery, puddings, biscuits, savoury snacks, and sugary beverages [11]. These foods are typically high in fats, salt and sugar (HFSS), and they fall outside the five core food groups [11] and are commonly promoted in prominent locations within supermarkets [15]. The modelled intervention would restrict unhealthy food placement in high-visibility areas such as end-of-aisles, checkouts, and store entrances.

### Comparator

Currently there are no restrictions on the placement of unhealthy foods and beverages in supermarkets, therefore the comparator was the simulation of the 2024 Australian population, aged 2-100 years, assuming no exposure to the intervention.

### Assessment of Benefits

**Fig. 1:**
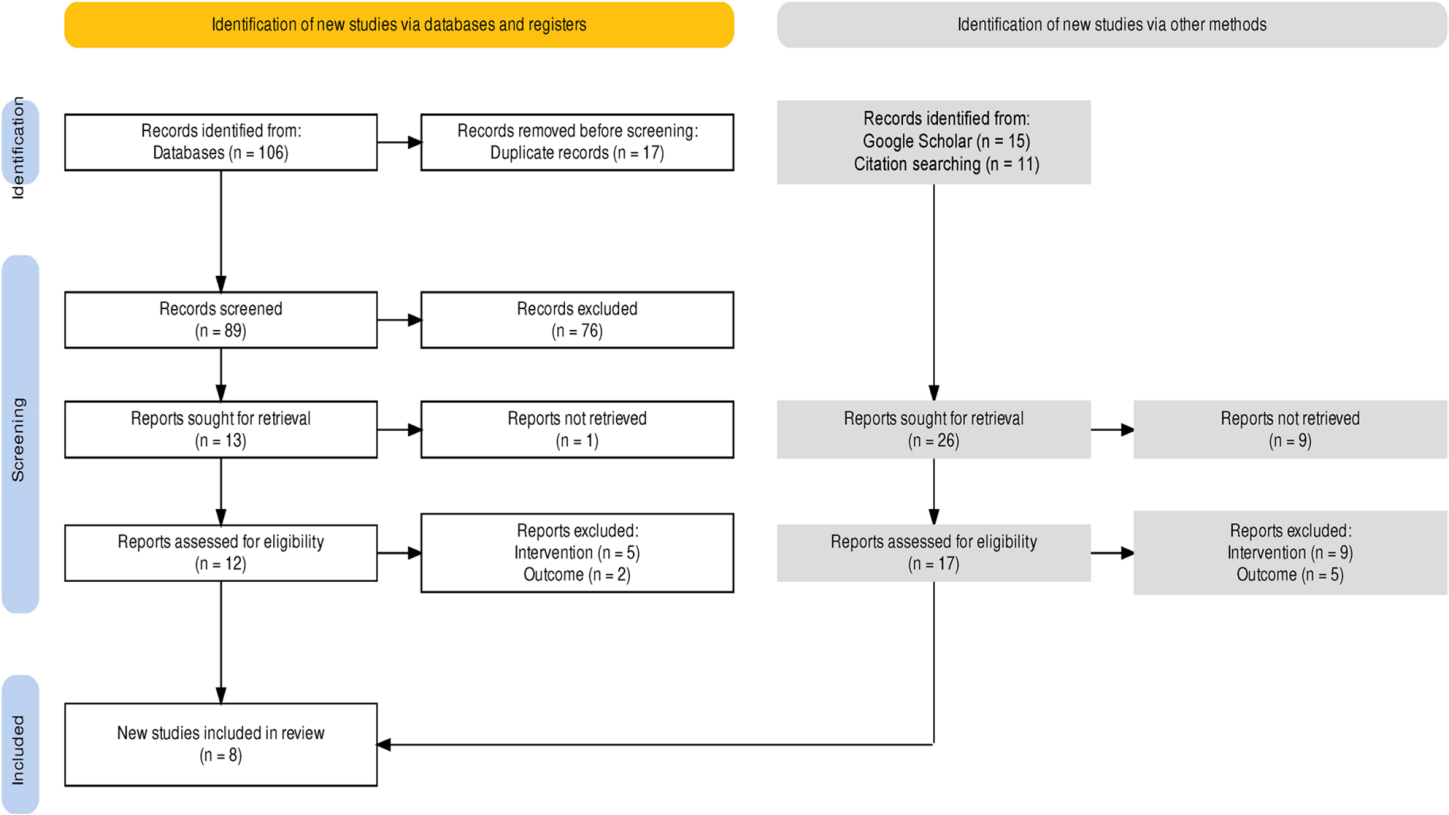
Flow chart of the study selection process

A pragmatic literature review was conducted to assess the effectiveness of the proposed intervention. Searches were performed on November 4, 2023, in four databases: APA PsychArticles, Journals@Ovid Medline^®^, Embase, and Health Management Information Consortium, supplemented by a Google Scholar search. The search strategy is detailed in (Supplementary File Table A1). Studies were included if they assessed the impact of unhealthy food placement restrictions (at checkouts, end-of-aisles, and store entrances) in retail settings such as supermarkets, grocery stores, and convenience stores. Studies were required to report outcomes such as purchasing data, dietary intake, or anthropometric measures (e.g., BMI). Studies were excluded if they exclusively targeted factors such as price, food labelling, portion size, or the placement and availability of healthy foods only (rather than the placement of unhealthy foods), or if their reporting focused solely on the availability of unhealthy food items or the space allocated to them without evaluating the impact of these restrictions on purchasing, diet or health outcomes. The first author (BO) screened all titles and abstracts against the eligibility criteria, conducted full-text reviews of potentially relevant studies, and extracted data into Microsoft Excel for synthesis and analysis. Data extraction was reviewed by all authors. A total of 132 papers were identified, 29 underwent full-text review, and 8 studies were included in the final synthesis, as detailed in Fig. 1 and Supplementary File 1 Table A3.

Among the included studies, two [12, 18] were selected due to their methodological rigour, relevance to real-world retail placement restrictions, and reporting of measurable changes in purchasing behaviour. Piernas et al. and Ejlerskov et al. were selected as they employed a non-randomised trial and natural experiment designs and evaluated the impact of the intervention using interrupted time series analyses of sales data, with appropriate comparison periods or control stores, to report the impact of placement restrictions on purchasing. The study by Piernas et al. [12] reported a statistically significant reduction in the seasonal increase in chocolate confectionery purchased in prominent locations in the intervention stores, compared to the control stores, (12% increase in intervention versus 31% in control stores). The study by Ejlerskov et al. [18] combined both longitudinal and cross-sectional analyses to demonstrate a sustained reduction (15.5% after 1 year) in purchases of the targeted unhealthy foods (sugary confectionery, chocolate, and crisps) following the implementation of restrictions at supermarket checkouts. The combined effect size derived from Ejlerskov et al. [18] and Piernas et al. [12] was used to calculate the weighted average reduction in mean energy intake of 17.25%. There is limited evidence on the impact of removing sugary drinks from prominent store locations, largely due to study limitations such as the absence of appropriate control groups, which makes it difficult to establish causal effects. As a result, the combined effect size was applied to both restricted foods and drinks in the analysis. The Piernas et al. [12] study didn’t specify which location restrictions were applied and only restrictions to checkouts were applied in the Ejlerskov et al. study [18]. The current modelling employs a conservative assumption that the proposed policy which limits the placement of restricted foods in all prominent locations including checkouts, end of aisles and store entrances has similar effectiveness to restrictions observed in the studies selected which applied restrictions to limited prominent locations.

### Estimating the effectiveness of the proposed intervention

**Fig. 2:**
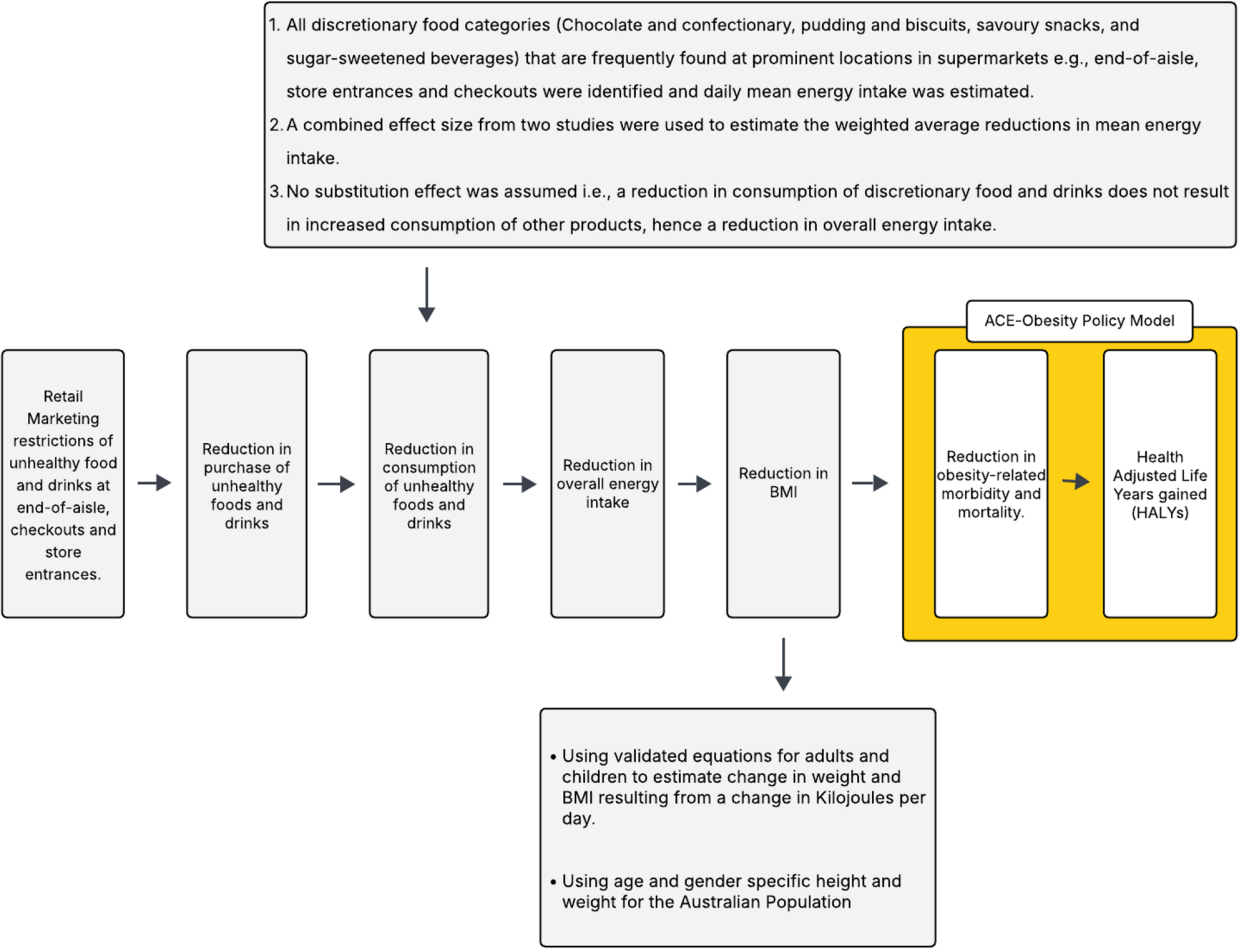
Assumptions involved in calculating the reduction in consumption of unhealthy food and drinks

The ideal evidence to inform cost-effectiveness modelling would be the effect on BMI derived directly from restricting the placement of unhealthy foods and drinks in prominent supermarket locations. However, due to lack of such evidence, a logic pathway (Fig. 2) to model the impact of the proposed intervention on population changes in energy intake, weight, and BMI was required and is detailed below.

### Impact on population energy intake and weight

The first step involved calculating the energy intake from restricted products at supermarkets impacted by the proposed intervention for both children (ages 2–17) and adults (18+) by 5-year age groups, across both genders. Energy intake from these products (chocolates and confectionery, puddings and biscuits, savoury snacks, and sugar-sweetened beverages) was adjusted to reflect the market share of the supermarkets impacted by the proposed intervention (82.4%) [21] and the percentage of chocolates purchased from Australian supermarkets (76%) [22]. The second step combined the immediate reduction in unhealthy food purchases observed after implementing the restrictions using the estimates from both Ejlerskov et al. [18] and Piernas et al. [12] (17.25%) to calculate the expected daily energy intake reduction in kilojoules (kJ) by age and sex, assuming the same percentage reduction in consumption with no compensatory consumption of other non-restricted products. The kJ reduction was converted to changes in body weight based on previously published equations for adults [23] and children [24]. Changes in weight were converted to changes in BMI using average Australian height and weight by sex and single-year age groups up to 19 years [25] and 5-year age groups up to 100 years [25].

### Cost of intervention implementation and monitoring

The costs of implementing the intervention were assessed from a limited societal perspective. This included costs to the government and retailers over the modelled time horizon. A bottom-up approach was used to estimate the cost and was based on the impact assessment done in the UK on restricting the placement of discretionary products at prominent locations in supermarkets [15]. The UK assessment was selected as a reference point due to its comprehensive evaluation of a similar policy intervention, comparable retail environment, and recent implementation experience. Staff time costs were valued using average wages for different occupations reported by the Australian Bureau of Statistics [26] and included labour on-costs (13%) and 17.5% leave loading. Costs were identified, measured and valued in Australian dollars (A$) and were adjusted to A$2024 values using the Consumer Price Index [27].

Costs accrued by government included the costs of enacting legislation in the Australian Parliament [28], cost of monitoring the intervention post implementation [29] and cost of a targeted media campaign to inform impacted businesses [30]. The cost of administration and monitoring were based on a cost-benefit analysis of a supermarket intervention undertaken by Ananthapavan et al. [29] It was assumed that approximately 7% of supermarkets would be checked by a government officer in the first three years to ensure policy compliance. After the initial familiarisation period (3 years), it was assumed that all supermarkets would be compliant, and the intervention would be in a steady state and therefore no further monitoring costs would be incurred.

The cost to the supermarket industry was categorised into transition and compliance costs. For the transition cost, it was assumed that the four major supermarkets [31] in Australia would implement the intervention involving a general manager at each of the supermarket head offices being responsible for regulation, implementation and providing information to the stores. This was estimated to take 12 h. To distribute information to the individual stores (totalling 4,105 stores [32–34], it was assumed that a retail manager and two stock control clerks would be tasked with familiarising themselves with the requirements of the legislation and executing the intervention at each store. The estimated time for each of these staff was assumed to be 3 h [15]. Other costs included the time required for the retailers to evaluate all food products according to the regulations, and this was assumed to happen at the head office level, allowing managers to distribute centrally compiled lists. It was assumed that the assessment would take approximately 5 to 10 min per product per supermarket chain, and this would apply to 79% of total products sold in supermarkets (21% are non-food items) [15]. Store layout changes to relocate the discretionary items to their new locations was assumed to require a retail manager and store control clerk at each store spending one day each (7.6 h), and 4 sales assistants (1.5 days) to rearrange the layout. Given the uncertainty of the time spent for replanning store layout, this was tested in the scenario analysis.

Ongoing product assessment costs were assumed to continue over the modelled period as new products enter the market continually. It was assumed that the General managers in each head office would spend a total of 5 to 10 min evaluating new food products against the restriction criteria [15]. The total number of new food products requiring assessment each year was calculated by applying the proportion of food products (79%) [21] to the number of new food products introduced at each supermarket chain per year [33, 35]. 

A recently published study in the UK reported that after one year of implementation of supermarket placement restrictions, approximately 30% of supermarket stores were non-compliant [36]. Based on this, it was assumed that 30% of the individual stores within the 7% of stores checked during policy monitoring would also be non-compliant. It was assumed that the percentage of non-compliant stores would reduce to 20% and 10% in the second and third year of policy implementation. Non-compliant stores would be required to pay a penalty of A$5,500 [37] (cost to retailers of non-compliance). The unit costs and resources used to implement the intervention are detailed in Supplementary File 3 Table A7.

### Cost-effectiveness modelling

A proportional, multi-state, life table Markov model (ACE-Obesity Policy model) was used to estimate the changes in long term health and associated healthcare cost [20]. The model was used to simulate the impact of population changes in BMI on the incidence, prevalence, morbidity, and mortality of nine obesity-related diseases: ischaemic heart disease, hypertensive heart disease, ischaemic stroke, diabetes, colorectal cancer, kidney cancer, breast cancer, endometrial cancer, and knee and hip osteoarthritis [20]. For each disease, the model included four health states: healthy, diseased, deceased due to the disease, and deceased due to other causes. Disability weights from the 2017 Global Burden of Disease Study [38] were applied to capture the impact of these states on health outcomes. The ICER was calculated as the ratio of the incremental net costs to the incremental health benefits of the intervention compared to status quo.

**Fig. 3:**
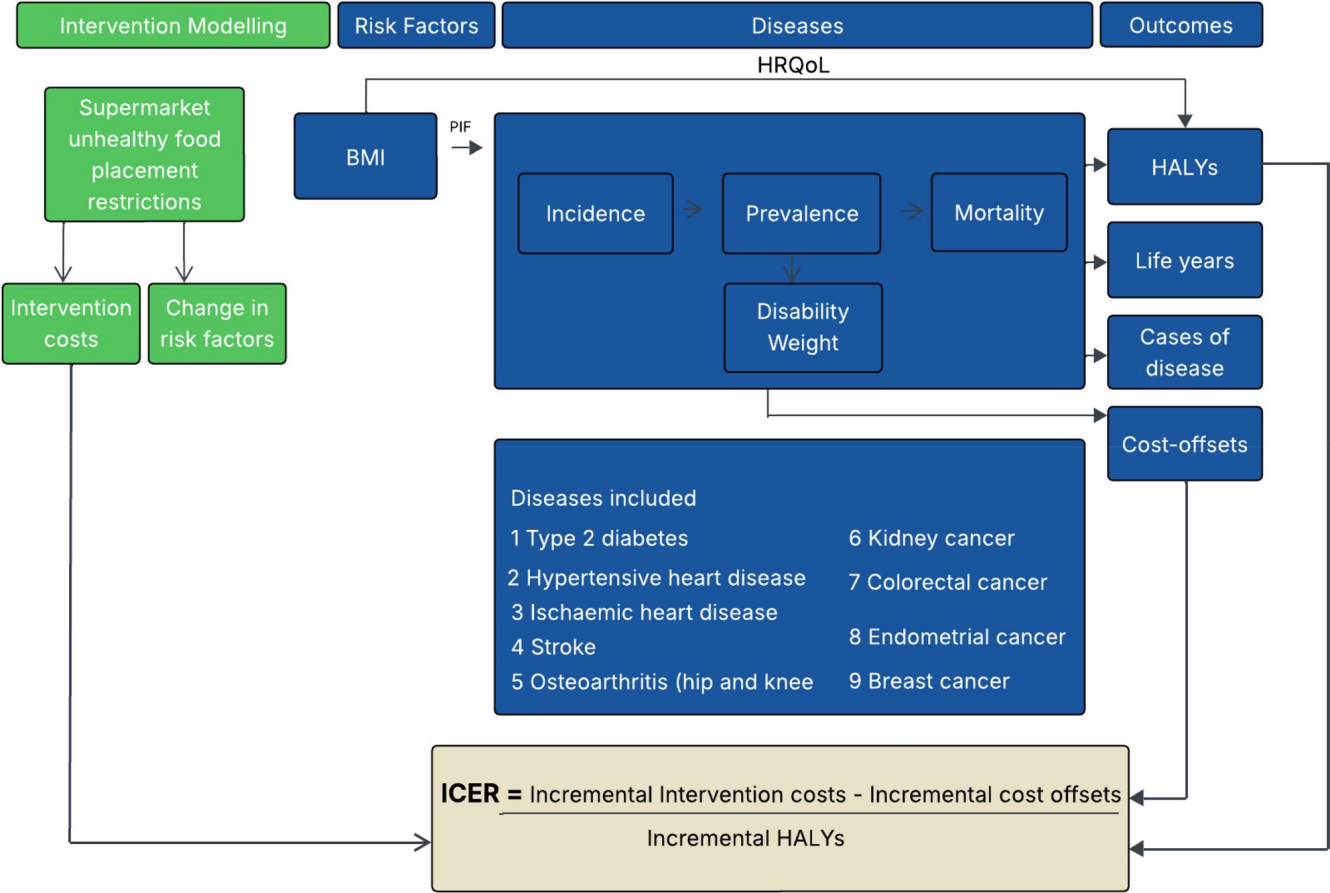
The ACE-Obesity Policy Model [20], © Deakin University 2018, reproduced with permission. BMI: Body Mass Index, PIF: Potential Impact Fraction, HRQoL: Health Related Quality of Life, HALYs: Health Adjusted Life Years, ICER: Incremental Cost-Effectiveness Ratio

Modelling was conducted using Microsoft Excel 2016. Both deterministic and PSA were performed to evaluate the robustness of model results and to assess the impact of uncertainties in key model inputs and assumptions. PSA was conducted through Monte Carlo simulation using Ersatz software (version 1.3) to incorporate parameter uncertainty. Each model input was assigned a probability distribution, and the PSA generated pairs of incremental HALYs and incremental costs by sampling values from these distributions. A total of 2,000 simulations were performed to assess the variability of the results. Prior to running the analysis, quality checks were conducted. This included verification of formulas and parameter linkages, consistency checks across outputs, and confirmation that results behaved as expected under deterministic and probabilistic analyses. Deterministic results were replicated prior to probabilistic analysis to ensure stability and coherence of model outputs. The ACE-Obesity Policy model structure is depicted in Fig. 3.

**Table 1:** Scenario Analysis

	Scenario Analysis	Rationale
1	7% Discount rate	Discount rate recommendations by the Australian Office of Impact Analysis were tested [39].
2	10% Discount rate	
3	Increased time spent for replanning store layout to 3 days	The model assumed a day, but the UK’s impact assessment [15] assumed it would take 3 days, which would increase the total intervention cost.
4	Restricting the impact of the policy to chocolates and confectionery food groups only	The strongest evidence for the effectiveness of this intervention is for restrictions on chocolate and confectionary.
5	10-year time horizon	Australian governments recommend testing economic analyses over shorter timeframes [39].

## Results

The intervention was estimated to reduce population daily energy intake, weight, and BMI across both genders (Table 2).

**Table 2:** Effectiveness of supermarket unhealthy food placement restrictions

Mean changes kJ intake (kJ)	Males	Females
	-83.41 (95% UI: -64.94; -106.05)	-56.29 (95% UI: -43.73; -70.76),
Mean changes in weight (kg)	-0.75 (95% UI: -0.59; -0.96 kg)	-0.52 (95% UI: -0.41; -0.66)
Mean changes in BMI (kg/m^2^)	-0.26 (95% UI: -0.21; -0.34).	-0.21 (95% UI: -0.17; -0.27).

*KJ* Kilojoules,* kg* Kilogram, *BMI* Body Mass Index, *kg/m2* Kilogram per meter square, *UI* Uncertainty intervals

### Costs

The total lifetime intervention cost was estimated at A$19.59 M (95% UI: A$16.49 M to A$24.62 M) (Table 3) with most of the cost (76%) being borne by the supermarket industry, A$14.94 M (95% UI: A$12.89 to A$17.60). Government costs were estimated to be A$4.66 M (95%UI: A$3.32 to A$7.35).

**Table 3:** Cost of Intervention Implementation (A$ Millions, with 95% UI)

Cost description	Sector			Total Cost
	Industry	Government	Healthcare	
One-off costs				
Transition costs	A$12.78 M (A$10.95 M; A$15.27 M)	A$2.51 M (A$2.37; A$2.65)		A$15.29 M (A$13.32 M; A$17.92 M)
Passing legislation		A$1.56 M (A$1.56 M; A$1.56 M)		A$1.56 M (A$1.56 M; A$1.56 M)
Media campaign		A$0.96 M (A$0.82 M; A$1.09 M)		A$0.96 M (A$0.82 M; A$1.09 M)
On-going				
Costs of handling complaints	A$1.86 M (A$0.71 M; 4.25 M)			A$1.86 M (A$0.71 M; 4.25 M)
Monitoring costs (year 1, 2 and 3)		A$0.74 M (A$0.28 M; A$1.67 M)		A$0.74 M (A$0.28 M; A$1.67 M)
Total Intervention costs	A$14.94 M (A$12.89 M; A$17.60 M)	A$4.66 M (A$3.32 M; A$7.35 M)		A$19.59 M (A$16.49 M; A$24.62 M)
Healthcare Costs			-A$4392.25 M (-A$3186.77 M; -A$5855.91 M)	
Total Net Cost *(Cost savings)*				-A$4411.81 M (- A$3206.01 M; -A$5874.69 M)

*A$* Australian dollars in 2024 values, *UI* Uncertainty Intervals, *M *Million. Negative costs represent cost savings

### Cost-Effectiveness Results

**Fig. 4:**
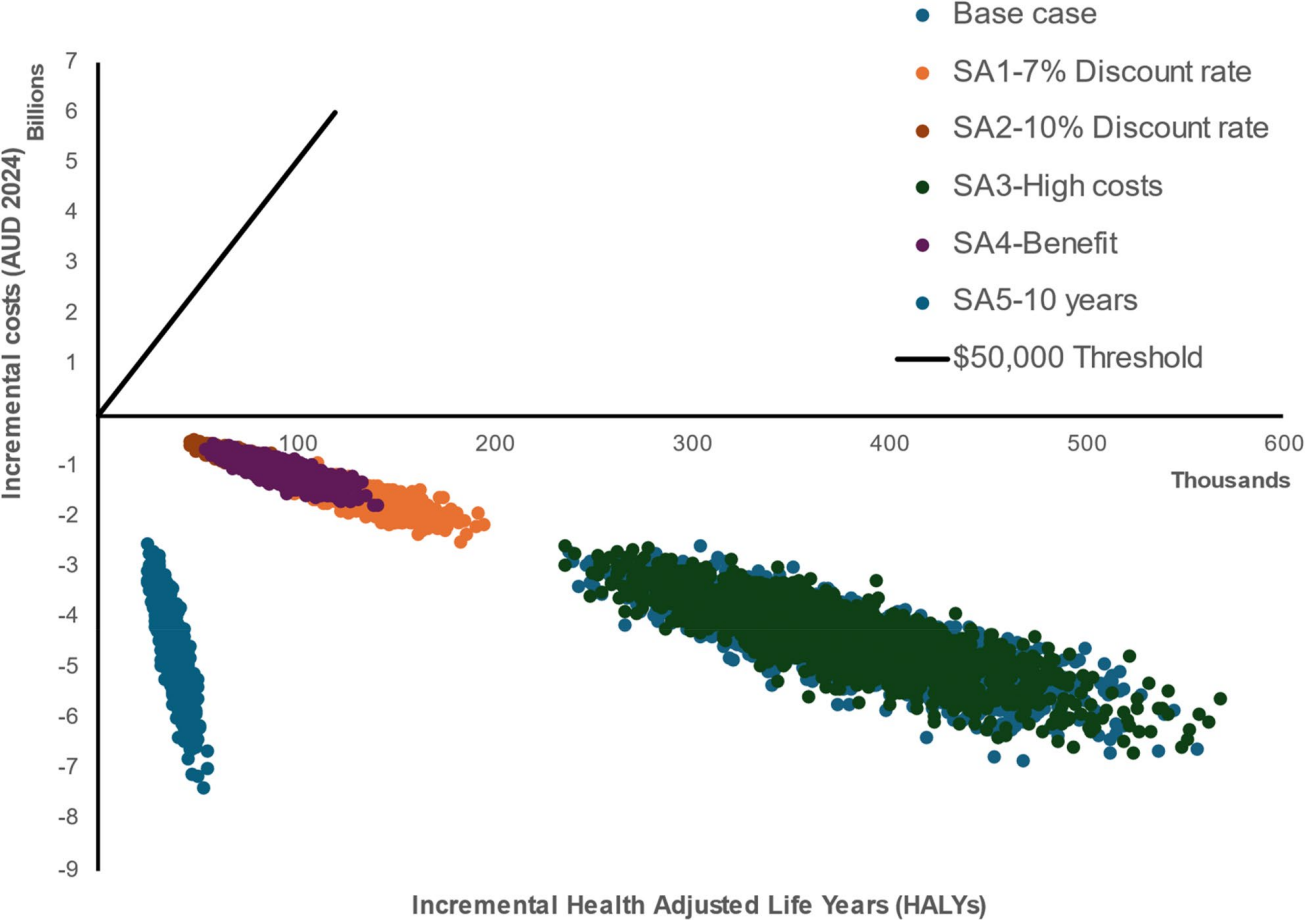
The Cost-effectiveness Plane (CEP). SA: Sensitivity Analysis

The primary analysis demonstrated that the intervention was dominant, delivering both improved health outcomes and reduced cost when compared to a ‘status quo’ comparator (Table 4). The intervention demonstrated all ICER iterations were positioned in the southeast quadrant of the Cost-Effectiveness Plane (Fig. 4), indicating that the intervention was consistently favourable in terms of value for money when compared to the status quo. The intervention generated significant health benefits of 0.37 M HALYs gained (95% UI: 0.28 M to 0.48 M). The financial impact was notably positive, with reduced health care costs of A$4,392.25 M (A$3,186.77 M to A$5,855.91 M) resulting in a net cost of -A$4,411.81 M (- A$3,206.01 M to -A$5,874.69 M).

**Table 4:** Primary analysis: cost effectiveness

Results	Mean Value (95% UI)
Total intervention cost	A$19.59 M (A$16.49 M; A$24.62 M)
Total healthcare costs	-A$4392.25 M (-A$3186.77 M; -A$5855.91 M)
Total net cost	-A$4411.81 M (- A$3206.01 M; -A$5874.69 M)
Total HALYs	0.37 M (0.28 M; 0.48 M)
ICER	Dominant (Dominant; Dominant)

*ICER* Incremental Cost−Effectiveness Ratio, *M* Million, *UI* Uncertainty Interval. Negative costs represent cost savings

### Sensitivity analysis

The intervention remained dominant across all sensitivity analyses conducted. (Table 5).

**Table 5:** Sensitivity analyses- Results (mean, 95% uncertainty interval)

Results	SA1- 7% Discount rate	SA 2–10% Discount rate	SA 3- Increased time to replanning store layout (3 days)	SA 4- Restricting the impact of the policy on only chocolates and confectionery food groups	SA 5–10-year time horizon
Mean change in BMI (Kg/m^2^) Males	-0.26 (-0.34; -0.21)			-0.05 (-0.06; -0.04)	-0.26 (-0.33; -0.21)
Mean change in BMI (Kg/m^2^) Females	-0.21 (-0.27; -0.17)			-0.06 (-0.08; -0.05)	-0.21 (-0.27; -0.16)
Total intervention costs	A$16.23 M (14.32;18.80)	A$15.96 M (14.04; 18.53)	A$27.46 M (24.02; 32.49)	A$ 19.59 M (16.44; 24.48)	A$18.01 M (15.06; 22.79)
Total healthcare costs	-A$1,549.41 M (-A$1126.29; -A$2036.81)	-A$880.74 M (-A$649.99; -A$1153.73)	-A$4,381.10 M (-A$3180.26; -A$5,832.60)	-A$1,111.49 M (-A$782.90; -A$1512.30)	-A$4,393.11 M (-A$3158.40; -A$5805.19)
Total net cost	-A$1,533.15 M (-A$1110.84; -A$2,022.02)	-A$864.83 M (-A$634.76; -A$1,137.27)	-A$4,353.55 M (-A$3155.88; -A$5,806.37)	-A$1,091.81 M (-A$765.35; -A$1492.11)	-A$4,375.10 M (-A$3136.05; -A$5789.08)
Total HALYs gained	0.12 M (0.09; 0.16)	0.07 M (0.06; 0.09)	0.37 M (0.28; 0.49)	0.09 M (0.07; 0.12)	0.03 M (0.03; 0.04)
ICER	Dominant (Dominant; Dominant)				

*SA* Sensitivity Analysis,* KJ* Kilojoules, *kg* Kilogram, *BMI* Body Mass Index, *kg/m2* Kilogram per meter square, *UI* Uncertainty intervals, Incremental Cost−Effectiveness Ratio; *M* Million, *A$* Australian dollar

## Discussion

This study has shown that placement restrictions on unhealthy food in supermarkets leads to substantial health gains and healthcare cost savings. These findings highlight the value of population-wide interventions as effective strategies for obesity prevention. The results of the sensitivity analyses reinforced these findings, consistently demonstrating the policy to be dominant across diverse scenarios. Modifying supermarket environments has significant potential to improve population health, given their wide population reach and the ability to scale interventions across multiple stores within large retail chains. In Australia, the grocery market is among the most concentrated globally, with four major chains, Woolworths, Coles, Aldi, and IGA [10] accounting for a combined market share of 82.4% [21]. This high concentration increases the reach and effectiveness of any changes made in the retail food environment [40]. The promotion, convenience, and affordability of unhealthy foods and drinks often overshadow healthier options, further complicating efforts to encourage better dietary choices [6, 9]. This study has shown that even modest changes in supermarkets have the capacity to induce meaningful dietary shifts at the population level [4]. This study is the first, to our knowledge, to model the cost-utility of retail marketing restrictions in Australian supermarkets. It contributes valuable evidence to existing literature and policy discussions, particularly in the context of Australia’s National Obesity Strategy [6] and the National Preventive Health Strategy which emphasises working with supermarket retailers as key policy areas [2, 12].

A major strength of this study is its policy relevance as the intervention strongly aligns with the objectives of the WHO, national Australian strategies, and an impact assessment conducted in the UK on restricting the promotion of HFSS food and drinks at prominent locations, which found that the policy delivered significant health benefits, cost savings, and resulted in a positive net present value [15]. Another strength of the study is the use of the ACE-Obesity Policy model, previously applied to assess the cost-effectiveness of 16 obesity prevention interventions across various settings [20]. Although the models have been updated over time, the consistent methodology allows for cautious comparisons. This suggests that restricting the placement of unhealthy food and drinks at prominent locations in Australian supermarkets could be a valuable addition to a suite of evidence-based policies for obesity prevention and public health improvement in Australia.

This study is not without limitations. Firstly, there was no direct evidence of policy impact on change in population BMI. The analysis therefore had to use conservative assumptions using a logic model. To address this uncertainty, sensitivity analysis was conducted in relation to the effect size used in the modelling. The lack of clear evidence on effectiveness is due to the difficulty with implementing high quality randomised controlled trials for population level policy interventions, and the lack of policy implementation globally. To our knowledge, only the UK has implemented similar regulations. Secondly, the effectiveness estimates informing behavioural change were derived from two studies, Piernas et al. and Ejlerskov et al. [12, 18]. These studies focused on unhealthy foods rather than beverages. While sugar-sweetened beverages were included in baseline discretionary energy intake, the application of a uniform proportional reduction across product categories assumes similar consumer responses to placement restrictions, which may not fully reflect category-specific purchasing behaviour. A third limitation is the nature of the studies included which did not allow for potential substitution effects. For example, it remains unclear whether people might compensate for reduced energy intake from the intervention by consuming other foods or healthier alternatives. Additionally, this analysis integrates parameters derived from multiple external sources and modelling assumptions. While this approach is standard in health economic and policy modelling, it introduces both structural and parameter uncertainty arising from differences in study designs and data sources. To address this, uncertainty was explicitly incorporated through PSA, which allows simultaneous variation across all model inputs. Additionally, the assumption that full compliance with the policy is achieved after three years may be optimistic for a national retail policy. Uncertainty around this assumption was explored by varying the proportion of non-compliant stores in years 1–3. Given the relatively small contribution of monitoring costs to total costs, alternative compliance assumptions are unlikely to materially affect the overall cost-effectiveness conclusions. The adoption of a limited societal perspective was also a limitation, as any impact on productivity and potential revenue losses to the food industry was not possible to model due to a lack of available data [19].

A key step that should precede the implementation of this policy is a qualitative assessment of implementation considerations. Unlike other interventions assessed as part of the ACE-Obesity Policy study [20], this analysis did not include such an assessment. The ACE methodology is distinguished by its consistent approach, which addresses the broader concern of decision-makers beyond formula-driven decision-making [40]. Therefore, a second stage should qualitatively examine significant implementation considerations, such as strength of evidence, equity, acceptability, feasibility, and sustainability, and present these alongside the cost-effectiveness results to aid decision-making [20].

Future research should focus on assessing the direct impact of the intervention on the outcomes of interest, this would offer a more robust measure of effectiveness compared to the more distal outcomes, such as changes in purchasing behaviour. This will require long-term monitoring data after policy implementation to demonstrate impacts on weight or BMI rather than the more distal outcome measure of changes in purchasing. The equity impacts of this policy were not modelled and the effectiveness of this policy across population groups may depend on differences in shopping patterns and other promotions within the store setting. Another economic analysis assessed that supermarket-based interventions related to labelling have a neutral equity impact [29]. This is a structural intervention that improves the healthiness of the food environment and therefore may have a positive equity impact [41]. Future research is required to assess the differential impact of this policy across socioeconomic and other relevant equity groups.

## Conclusions

This study suggests that restricting the placement of unhealthy foods in prominent supermarket locations in Australia may lead to meaningful health gains and represent a potentially cost-effective policy option. The findings highlight the role that policy-driven retail interventions could play in shaping healthier food environments as part of broader efforts to address poor diet and obesity. The results indicate that placement restrictions could be a valuable component of a multi-pronged strategy to improve population diets and health.

## Supplementary Information

Supplementary Material 1.

## Data Availability

All the data generated or analysed during this study are included in this published article and its supplementary information flies.

## Abbreviations

NCDs: Non-Communicable Diseases
USA: United States of America
WHO: World Health Organisation
ADG: Australian Dietary Guidelines
BMI: Body Mass Index
ACE: Assessing Cost Effectiveness
ICER: Incremental Cost-Effectiveness Ratio
PSA: Probabilistic Sensitivity Analysis
UI: Uncertainty Intervals
HALYs: Health Adjusted Life Years
HFSS: High in Fats, Salts and Sugar
KJ: Kilojoules
UK: United Kingdom
A$: Australian dollars
SA: Sensitivity Analysis
Kg: Kilogram
Kg/m^2^: Kilogram per meter square
M: Million
HRQoL: Health Related Quality of Life
CEP: Cost-Effectiveness Plane
