# Supplementary material for: The cost-effectiveness of placement restrictions of unhealthy food and drinks at end of aisles and checkouts in Australian supermarkets
Source: Nutr J. 2026 Mar 13;25:56. doi: 10.1186/s12937-026-01307-9 (PMC13130497; doi:10.1186/s12937-026-01307-9)
Supplement: Supplementary file 1 — Supplementary Material 1. [file 12937_2026_1307_MOESM1_ESM.docx]

**APPENDICES**

**Appendix 1**

**Table A1: Search Strategy**

| **S/N** | **Searches** | **Results** |
| --- | --- | --- |
| 1 | sugar or sweetened beverages or confectionery or chocolate | 457633 |
| 2 | (((unhealthy food or discretionary food or High in fat, sugar) and salt food) or HFSS food). | 141 |
| 3 | (diet or dietary intake or energy intake). | 2091953 |
| 4 | (food or fruit or nutrient or nutritious). | 3315401 |
| 5 | (nutrient sales or food purchasing). | 2617 |
| 6 | (food preference or food supply or food industry). | 126614 |
| 7 | supermarket | 16181 |
| 8 | (grocery store or retail store or in store). | 117452 |
| 9 | (prominent locations or end-of-aisle or checkouts or store entrance). | 622 |
| 10 | (placement or promotion or restriction or display) | 3396825 |
| 11 | (health or obesity or overweight or body mass index or BMI) | 14551571 |
| 12 | marketing restrictions | 795 |
| 13 | 1 or 2 | 457644 |
| 14 | 3 or 4 or 5 or 6 | 4696283 |
| 15 | 13 and 14 | 174075 |
| 16 | 7 or 8 or 9 or 10 | 3490468 |
| 17 | 15 and 16 | 40893 |
| 18 | 15 or 16 | 3623650 |
| 19 | 11 and 12 and 18 | 465 |
| 20 | retail marketing strategies | 29 |
| 21 | 19 or 20 | 493 |
| 22 | 11 and 21 | 701 |
| 23 | 13 or 14 or 16 | 7822863 |
| 24 | 13 and 14 and 16 and 23 | 40893 |
| 25 | 9 and 10 and 24 | 106 |
| 26 | limit 25 to English language [Limit not valid in Journals@Ovid, HMIC; records were retained] | 106 |
| 27 | remove duplicates from 26 using Ovid | 89 |

**Table A2: PICOS for pragmatic Literature Review**

| **PICOS** | **Inclusion criteria** | **Exclusion criteria** |
| --- | --- | --- |
| Population | N/A | N/A |
| Intervention | Restricting placement of discretionary foods at prominent locations to influence consumer nutrition/diet. | Studies that focus solely on price, food labelling portion size, placement or availability of healthy foods.  Inadequate description of in-store placement measures or strategies. |
| Comparator | Current practice/status quo | Anything else |
| Outcome | Store sales data, self-reported food purchase data, consumer food consumption, dietary intake and physical measures such as BMI | Process measures, customer or staff knowledge/awareness/attitude, customers' intended purchasing behaviours. |
| Study design | Intervention studies, Observation studies, Systematic reviews. |  |
| Setting | Supermarkets, grocery stores, convenience stores, checkouts, end-of-aisles, store entrances. | Non-permanent locations (outdoor markets, pop-up stalls), speciality food stores, cafeterias. |
| Limits | English language publications | Non-English language publications |

**Table A3: Study Characteristics of papers restrict placement of unhealth food +/- promoting health foods)**

| Study | Country | Participants/Setting | Intervention type | Comparator | Intervention design | Intervention description | Intervention duration | Outcome | Data collection tools | Results |
| --- | --- | --- | --- | --- | --- | --- | --- | --- | --- | --- |
| (Piernas, Harmer and Jebb, 2022) | UK | 34 intervention stores located in 2 London boroughs and 151 matched control stores located elsewhere in the UK. | Placement | 151 matched controlled stores located elsewhere in the UK. | Non-randomized controlled intervention study with interrupted time series (ITS) analysis | The intervention aimed to reduce the excess availability of seasonal chocolate confectionery by eliminating free-standing promotional display units from prominent locations and replacing seasonal confectionery (n=178) positioned at the end of aisles with alternative products | 1 January 2018- 24 November 2019 (23 months) | Primary outcome measure: Store level weekly sales data (units, weight, and value) for the whole category of seasonal chocolate confectionery.  Secondary outcome measures: nutrient data (i.e., total energy, sugar, fibre, saturated fat, and total fat) from all food-related sales. | Experiments | During the pre-intervention baseline period, there were no notable variances in sales (units, weight, and value) of all chocolate confectionery between intervention and control stores. Following the implementation of the intervention, intervention stores experienced a decrease in the seasonal rise of confectionery sales (units) compared to control stores (+5% versus +18%; P<0.001). Similar effects were observed in terms of weight (grams) (+12% versus +31%; P<0.001) and value (GBP) (-3% versus +10%; P<0.001). |
| (DHSC, 2020) | UK | Retail stores and businesses in the UK. | Placement | Not reported | Not stated | End placement of HFSS foods and drink items which contribute significant sugar and calories to children’s diets and are of most concern for childhood obesity at store entrances, checkouts and end-of-aisles in the retail sector excluding small and micro businesses. | Not specified | HFSS and Non-HFSS Calories consumed and Net calorie reduction | Secondary data collection i.e., online databases | 4.9% reduction to the HFSS calories consumed and a 0.7% increase in non-HFSS calories consumed, given an overall net reduction in calories consumed |
| (Vogel et al., 2021) | UK | 3 discount supermarket control and intervention stores in England. Women customers aged 18 to 45 years with loyalty cards were assigned to the intervention (n=62) | Placement | Three control stores matched on store sales, customer profiles and neighbourhood deprivation. Women customers aged 18 to 45 years with loyalty cards were assigned to the control (n = 88) | A prospective matched controlled cluster trial with 2 intervention components | i) Fresh fruits and vegetables sections were relocated to store entrances, replacing smaller displays previously positioned at the back. Additionally, frozen vegetables were moved to the entrance aisle.  ii) Confectionery was removed from checkouts and aisle ends located opposite | 6 months | Effects on store-level sales, household-level purchasing and dietary behaviours | Experiments, Interview-administered telephone questionnaires. | Increase in store-level sales of fruits and vegetables were greater in intervention stores than predicted at 3 (1.71 (SDs) (95% CI 0.45, 2.96), P= 0.01) and 6 months follow-up (2.42 SDs (0.22, 4.62), P= 0.03), equivalent to approximately 6,170 and approximately 9,820 extra portions per store per week.  The proportion of purchasing fruits and vegetables per week rose among intervention participants at 3 and 6 months compared to control participants (0.2% versus -3.0%, P= 0.22; 1.7% versus -3.5%, P= 0.05, respectively).  Store sales of confectionery were lower in intervention stores than predicted at 3 (-1.05 SDs (-1.98, -0.12), P= 0.03) and 6 months (-1.37 SDs (-2.95, 0.22), P= 0.09), equivalent to approximately 1,359 and approximately 1,575 fewer portions per store, per week. |
| (Fildes et al; 2022) | England | UK Tesco Express convenience  stores in England | Product placement, availability | Purchase of less healthy foods in 2014 before introducing the healthy checkouts initiative. | A pre-post study | Tesco Express convenience stores implemented a healthy checkouts initiative; products high in fat, salt or sugar were removed from in-queue areas. | 8 weeks (May 2014 to July 2015) | Sales of less healthy products- Mean overall spend and proportion of total spend on less healthy foods. | Experiments | Mean overall spend increased in 2015 compared with 2014 (£666 079.70 [SD 406 385.00] vs. £653 786.59 [SD 447 580.77]; p < 0.001).  The proportion of total spending on less healthy foods decreased in 2015 versus 2014 (8.03% [SD 2.07] vs. 8.21% [SD 2.17]; p < 0.001).  Confectionery accounted for the largest proportion of less healthy product spend, showing the biggest reduction (3.91% [SD 1.16] in 2015 vs 4.12% [SD 1.24] in 2014; p < 0.001) |
| (Ejlerskov et al., 2018) | UK | Nine UK supermarkets (Aldi, Asda, Co-op, Lidl, M&S, Morrisons, Sainsbury's, Tesco, and Waitrose). | P  Product placement, availability | Three UK supermarkets did not implement a checkout food policy between 2013 and 2017. | Natural experimental evaluated using interrupted time series analyses of purchases | Six supermarkets implemented a checkout food policy between 2013 and 2017 and were designated as intervention stores, while the remaining stores were considered comparators | 4 years (2013 to 2017) | The weighted average percentage change in purchases of common checkout foods. | Experiments | The introduction of a checkout food policy led to an immediate reduction in purchases of common checkout foods by 157,000 (72,700-242,800) packages per percentage market share, representing a 17.3% reduction. This reduction was sustained at one year, with 185,100 (121,700-248,500) fewer packages bought per four weeks per percentage market share, equivalent to a 15.5% decrease. |
| (Adams et al; 2017) | Denmark | Five volunteering supermarket chains in Denmark where shelf space management intervention (SSMI) was implemented | Product placement, availability | Five volunteering supermarkets from the same chain as the intervention stores and within the same geographical locations in Denmark without SSMI. | Experimental controlled trial | A shelf space management intervention was conducted in five Danish stores representing different geographical locations in Denmark. Five other stores from the same chain with similar geographical locations were selected as control stores. A planogram was used to categorize dairy products into either red, green and yellow colour indicating high, low and medium energy density product, with the aim to promote products with relatively lower caloric content by making them easily accessible on shelves. | 10 weeks; 5 weeks of baseline period without intervention and 5 weeks intervention period. | Mean weekly sales of dairy products in terms of calories and kroners turnover | Experiments | The control store had a smaller decrease in mean weekly calorie turnover (-5.62 Kcal) compared to the intervention store (-7.91 Kcal), with a difference in difference of (-2.29)  A reduction in mean weekly sales revenue after the intervention with a reduction of (-36.87 1000 DKK/ product per store) in mean weekly sales revenue compared to the (-41.28 1000 DKK/ product per store) in the intervention store and a difference in difference of (-4.41) |

**Appendix 2: Effectiveness**

**Table A4: Weighted Mean Energy Intake Reductions per day (kj) for females and Males aged 2-17, 18+**

| **Males** | **Mean energy reduction after Intervention Implementation** | **Females** | **Mean energy reduction after Intervention Implementation** |
| --- | --- | --- | --- |
| Mean energy intake reductions per day (kj) males 2-5 | - 60.10 | Mean energy intake reductions per day (kj) females 2-5 | - 53.85 |
| Mean energy intake reductions per day (kj) males 6-10 | - 101.11 | Mean energy intake reductions per day (kj) females 6-10 | - 77.53 |
| Mean energy intake reductions per day (kj) males 11-17 | - 123.46 | Mean energy intake reductions per day (kj) females 11-17 | - 86.94 |
| Mean energy intake reductions per day (kj) males 18-24 | - 119.59 | Mean energy intake reductions per day (kj) females 18-24 | - 73.79 |
| Mean energy intake reductions per day (kj) males 25-29 | - 97.02 | Mean energy intake reductions per day (kj) females 25-29 | - 63.55 |
| Mean energy intake reductions per day (kj) males 30-34 | - 102.88 | Mean energy intake reductions per day (kj) females 30-34 | - 62.81 |
| Mean energy intake reductions per day (kj) males 35-39 | - 84.88 | Mean energy intake reductions per day (kj) females 35-39 | - 55.37 |
| Mean energy intake reductions per day (kj) males 40-44 | - 85.06 | Mean energy intake reductions per day (kj) females 40-44 | - 54.89 |
| Mean energy intake reductions per day (kj) males 45-49 | - 82.91 | Mean energy intake reductions per day (kj) females 45-49 | - 54.03 |
| Mean energy intake reductions per day (kj) males 50-54 | - 69.17 | Mean energy intake reductions per day (kj) females 50-54 | - 49.69 |
| Mean energy intake reductions per day (kj) males 55-59 | - 64.61 | Mean energy intake reductions per day (kj) females 55-59 | - 40.03 |
| Mean energy intake reductions per day (kj) males 60-64 | - 65.08 | Mean energy intake reductions per day (kj) females 60-64 | - 38.19 |
| Mean energy intake reductions per day (kj) males 65-69 | - 43.15 | Mean energy intake reductions per day (kj) females 65-69 | - 37.16 |
| Mean energy intake reductions per day (kj) males 70-100 | - 48.39 | Mean energy intake reductions per day (kj) females 70-100 | - 39.72 |
| Mean energy intake reductions per day (kj) males Weighted average, aged 2-17 | - 101.36 | Mean energy intake reductions per day (kj) females Weighted average, aged 2-17 | -76.10 |
| Mean energy intake reductions per day (kj) males Weighted average, aged 18+ | - 78.91 | Mean energy intake reductions per day (kj) females Weighted average, aged 18+ | -51.70 |

**Table A5: Intervention Effectiveness Result- Change in Consumption, Energy Intake and Body Weight**

| **Retail restrictions change in weight-Males** | **kg** | **Retail restrictions change in weight-Females** | **kg** | **Retail restrictions change in BMI-Males** | **BMI** | **Retail restrictions change in BMI-Females** | **BMI** |
| --- | --- | --- | --- | --- | --- | --- | --- |
| Retail restrictions weight males-0 | NA | Retail restrictions weight females-0 | NA | Retail restrictions BMI males-0 | NA | Retail restrictions BMI females-0 | NA |
| Retail restrictions weight males-1 | NA | Retail restrictions weight females-1 | NA | Retail restrictions BMI males-1 | NA | Retail restrictions BMI females-1 | NA |
| Retail restrictions weight males-2 | - 0.26 | Retail restrictions weight females-2 | - 0.26 | Retail restrictions BMI males-2 | - 0.26 | Retail restrictions BMI females-2 | -0.26 |
| Retail restrictions weight males-3 | - 0.26 | Retail restrictions weight females-3 | - 0.26 | Retail restrictions BMI males-3 | - 0.26 | Retail restrictions BMI females-3 | -0.26 |
| Retail restrictions weight males-4 | - 0.26 | Retail restrictions weight females-4 | - 0.26 | Retail restrictions BMI males-4 | - 0.26 | Retail restrictions BMI females-4 | -0.26 |
| Retail restrictions weight males-5 | - 0.26 | Retail restrictions weight females-5 | - 0.26 | Retail restrictions BMI males-5 | - 0.18 | Retail restrictions BMI females-5 | -0.18 |
| Retail restrictions weight males-6 | - 0.46 | Retail restrictions weight females-6 | - 0.39 | Retail restrictions BMI males-6 | - 0.32 | Retail restrictions BMI females-6 | -0.27 |
| Retail restrictions weight males-7 | - 0.48 | Retail restrictions weight females-7 | - 0.42 | Retail restrictions BMI males-7 | - 0.33 | Retail restrictions BMI females-7 | -0.29 |
| Retail restrictions weight males-8 | - 0.50 | Retail restrictions weight females-8 | - 0.44 | Retail restrictions BMI males-8 | - 0.25 | Retail restrictions BMI females-8 | -0.21 |
| Retail restrictions weight males-9 | - 0.53 | Retail restrictions weight females-9 | - 0.47 | Retail restrictions BMI males-9 | - 0.26 | Retail restrictions BMI females-9 | -0.23 |
| Retail restrictions weight males-10 | - 0.56 | Retail restrictions weight females-10 | - 0.50 | Retail restrictions BMI males-10 | - 0.28 | Retail restrictions BMI females-10 | -0.24 |
| Retail restrictions weight males-11 | - 0.73 | Retail restrictions weight females-11 | - 0.60 | Retail restrictions BMI males-11 | - 0.36 | Retail restrictions BMI females-11 | -0.29 |
| Retail restrictions weight males-12 | - 0.78 | Retail restrictions weight females-12 | - 0.65 | Retail restrictions BMI males-12 | - 0.28 | Retail restrictions BMI females-12 | -0.26 |
| Retail restrictions weight males-13 | - 0.83 | Retail restrictions weight females-13 | - 0.70 | Retail restrictions BMI males-13 | - 0.30 | Retail restrictions BMI females-13 | -0.28 |
| Retail restrictions weight males-14 | - 0.89 | Retail restrictions weight females-14 | - 0.77 | Retail restrictions BMI males-14 | - 0.32 | Retail restrictions BMI females-14 | -0.30 |
| Retail restrictions weight males-15 | - 0.97 | Retail restrictions weight females-15 | - 0.85 | Retail restrictions BMI males-15 | - 0.35 | Retail restrictions BMI females-15 | -0.33 |
| Retail restrictions weight males-16 | - 1.05 | Retail restrictions weight females-16 | - 0.94 | Retail restrictions BMI males-16 | - 0.34 | Retail restrictions BMI females-16 | -0.36 |
| Retail restrictions weight males-17 | - 1.16 | Retail restrictions weight females-17 | - 1.07 | Retail restrictions BMI males-17 | - 0.37 | Retail restrictions BMI females-17 | -0.40 |
| Retail restrictions weight males-18-24 | - 1.20 | Retail restrictions weight females-18-24 | - 0.74 | Retail restrictions BMI males-18-24 | - 0.38 | Retail restrictions BMI females-18-24 | -0.28 |
| Retail restrictions weight males- 25-29 | - 0.97 | Retail restrictions weight females- 25-29 | - 0.64 | Retail restrictions BMI males-25-29 | - 0.31 | Retail restrictions BMI females-25-29 | -0.24 |
| Retail restrictions weight males - 30-34 | - 1.03 | Retail restrictions weight females - 30-34 | - 0.63 | Retail restrictions BMI males-30-34 | - 0.33 | Retail restrictions BMI females-30-34 | -0.24 |
| Retail restrictions weight males- 35-39 | - 0.85 | Retail restrictions weight females- 35-39 | - 0.55 | Retail restrictions BMI males-35-39 | - 0.27 | Retail restrictions BMI females-35-39 | -0.21 |
| Retail restrictions weight males - 40-44 | - 0.85 | Retail restrictions weight females - 40-44 | - 0.55 | Retail restrictions BMI males - 40-44 | - 0.28 | Retail restrictions BMI females - 40-44 | -0.21 |
| Retail restrictions weight males - 45-49 | - 0.83 | Retail restrictions weight females - 45-49 | - 0.54 | Retail restrictions BMI males- 45-49 | - 0.27 | Retail restrictions BMI females- 45-49 | -0.20 |
| Retail restrictions weight males - 50-54 | - 0.69 | Retail restrictions weight females - 50-54 | - 0.50 | Retail restrictions BMI males - 50-54 | - 0.22 | Retail restrictions BMI females - 50-54 | -0.19 |
| Retail restrictions weight males - 55-59 | - 0.65 | Retail restrictions weight females - 55-59 | - 0.40 | Retail restrictions BMI males - 55-59 | - 0.21 | Retail restrictions BMI females - 55-59 | -0.16 |
| Retail restrictions weight males - 60-64 | - 0.65 | Retail restrictions weight females - 60-64 | - 0.38 | Retail restrictions BMI males - 60-64 | - 0.22 | Retail restrictions BMI females - 60-64 | -0.15 |
| Retail restrictions weight males - 65-69 | - 0.43 | Retail restrictions weight females - 65-69 | - 0.37 | Retail restrictions BMI males - 65-69 | - 0.15 | Retail restrictions BMI females - 65-69 | -0.15 |
| Retail restrictions weight males - 70-100 | - 0.48 | Retail restrictions weight females - 70-100 | - 0.40 | Retail restrictions BMI males - 70-100 | - 0.16 | Retail restrictions BMI females - 70-100 | -0.16 |
| Retail restrictions weight males -Weighted average - children and adolescent 2-17 years | - 0.63 | Retail restrictions weight females -Weighted average - children and adolescent 2-17 years | - 0.56 | Retail restrictions BMI males -Weighted average - children and adolescent 2-17 years | - 0.30 | Retail restrictions BMI females -Weighted average - children and adolescent 2-17 years | - 0.28 |
| Retail restrictions weight males -Weighted average - adults 18+ years | - 0.79 | Retail restrictions weight females -Weighted average - adults 18+ years | - 0.52 | Retail restrictions BMI males -Weighted average - adults 18+ years | - 0.26 | Retail restrictions BMI females -Weighted average - adults 18+ years | -0.20 |

**Appendix 3: Place-based Restriction Costing**

**Table A6: Parameters (reference year: 2024)**

|  |  |
| --- | --- |
| Default work-related labour costs per hour | 48.67 |
| Non-wage labour on-costs and overhead costs (Source: OIA) | 1.75 |
| Work hours in a week (based on Fair Work Ombudsman) | 38 |
| Number of days worked in a week | 5 |
| Work hours in a day | 7.6 |
| Default leisure costs per hour | 37 |
| Number of weeks in a year | 52 |
| Total number of supermarket chains | 4.00 |
| Total number of supermarket stores | 4,105.00 |

**Table A7: Cost Estimate**

| **1** | **Regulatory burden** |  | **Mean /Median / Adjustments (as reported)** | **Mean/Median (2024)** | **SE** | **MIN/LL** | **MAX/UL** | **alpha** | **Beta** | **Dist** | **Ersatz** | **Eroutput** |
| --- | --- | --- | --- | --- | --- | --- | --- | --- | --- | --- | --- | --- |
|  | **A.** | **Supermarkets, convenient stores, and fuel retailing stores_START-UP** |  |  |  |  |  |  |  |  |  |  |
|  | **A.1** | **Familiarisation costs, assessment and implementation (start-up)** | **Mean /Median / Adjustments (as reported)** | **Mean/Median (2024)** | **SE** | **MIN/LL** | **MAX/UL** | **alpha** | **Beta** | **Dist** | **Ersatz** | **Eroutput** |
|  |  | HO: General manager |  |  |  |  |  |  |  |  |  |  |
|  |  | *Adjusted by non-wage labour and overhead costs* |  | 179.80 |  |  |  |  |  |  |  |  |
|  |  | Number of staff per HO | 1.00 |  |  |  |  |  |  |  |  |  |
|  |  | Time spent on new regulation familiarisation (hrs) |  | 12.00 |  | 8.00 | 16.00 |  |  | Pert | 12.00 |  |
|  |  | Total time spent on familiarisation, assessment and implementation (start-up) (hours/store) | 12.00 |  |  |  |  |  |  |  |  | 12.00 |
|  |  | ***Total familiarisation costs, assessment and implementation*** | ***8,630.28*** |  |  |  |  |  |  |  |  | 8,630.28 |
|  | **A.2** | **Costs of distributing information to individual stores** | **Mean /Median / Adjustments (as reported)** | **Mean/Median (2024)** | **SE** | **MIN/LL** | **MAX/UL** | **alpha** | **Beta** | **Dist** | **Ersatz** | **Eroutput** |
|  |  | HO: General manager |  |  |  |  |  |  |  |  |  |  |
|  |  | *Adjusted by non-wage labour and overhead costs* |  | 179.80 |  |  |  |  |  |  |  |  |
|  |  | Number of staff per HO |  | 1.00 |  |  |  |  |  |  |  |  |
|  |  | Time spent on information distribution (hours) |  | 1.00 |  | 0.80 | 1.20 |  |  | Pert | 1.00 |  |
|  |  |  |  |  |  |  |  |  |  |  |  |  |
|  |  | Retail/Store Manager |  |  |  |  |  |  |  |  |  |  |
|  |  | *Adjusted by non-wage labour and overhead costs* |  | 71.15 |  |  |  |  |  |  |  |  |
|  |  | Number of staff per store |  | 1.00 |  |  |  |  |  |  |  |  |
|  |  | Time spent on information distribution (hours) |  | 1.00 |  | 0.80 | 1.20 |  |  | Pert | 1.00 |  |
|  |  | Stock Control Clerk |  |  |  |  |  |  |  |  |  |  |
|  |  | *Adjusted by non-wage labour and overhead costs* |  | 55.75 |  |  |  |  |  |  |  |  |
|  |  | Number of staff per store |  | 2.00 |  | 1.60 | 2.40 |  |  | Pert | 2.00 |  |
|  |  | Time spent on information distribution (hours) |  | 1.00 |  | 0.80 | 1.20 |  |  | Pert | 1.00 |  |
|  |  | Total time spent on information distribution (hours/store) | 3.00 |  |  |  |  |  |  |  |  | 3.00 |
|  |  | ***Total costs of distributing information to individual stores*** | ***750,530.60*** |  |  |  |  |  |  |  |  | 750,530.60 |
|  | **A.3** | **Costs of product assessment (start-up costs, 1st year)** | **Mean /Median / Adjustments (as reported)** | **Mean/Median (2024)** | **SE** | **MIN/LL** | **MAX/UL** | **alpha** | **Beta** | **Dist** | **Ersatz** | **Eroutput** |
|  |  | General manager |  |  |  |  |  |  |  |  |  |  |
|  |  | *Adjusted by non-wage labour and overhead costs* |  | 179.80 |  |  |  |  |  |  |  |  |
|  |  | Number of staff per chain |  | 1.00 |  |  |  |  |  |  |  |  |
|  |  | Time spent on product assessment per product | 0.13 |  |  | 0.08 | 0.17 |  |  | Pert | 0.13 |  |
|  |  | Total time spent on product assessment (hours/chain/product) | 0.13 |  |  |  |  |  |  |  |  | 0.13 |
|  |  | *Supermarket chains:* | **Mean /Median / Adjustments (as reported)** | **Mean/Median (2024)** | **SE** | **MIN/LL** | **MAX/UL** | **alpha** | **Beta** | **Dist** | **Ersatz** | **Eroutput** |
|  |  | Number of products stocked at |  |  |  |  |  |  |  |  |  |  |
|  |  | *Woolworths* | *20,000* |  |  | *16,000.00* | *24,000.00* |  |  | *Pert* | *20,000.00* |  |
|  |  | *Coles* | *25,000* |  |  | *20,000.00* | *30,000.00* |  |  | *Pert* | *25,000.00* |  |
|  |  | *Aldi* | *1,350* |  |  | *1,080.00* | *1,620.00* |  |  | *Pert* | *1,350.00* |  |
|  |  | *IGA* | *10,958* |  | *1,890.00* |  |  | 33.62 | 325.98 | *Gamma* | *10,958.00* |  |
|  |  | *Total number of products stocked at supermarkets* | *57,308* |  |  |  |  |  |  |  |  | 57,308.00 |
|  |  | Proportion of food products at supermarkets | 79.00% |  |  | 0.63 | 0.95 |  |  | Pert | 0.79 | 0.79 |
|  |  | No. of food products undergoing product assessment |  |  |  |  |  |  |  |  |  |  |
|  |  | *Woolworths* | *15,800* |  |  |  |  |  |  |  |  |  |
|  |  | *Coles* | *19,750* |  |  |  |  |  |  |  |  |  |
|  |  | *Aldi* | *1,067* |  |  |  |  |  |  |  |  |  |
|  |  | *IGA* | *8,657* |  |  |  |  |  |  |  |  |  |
|  |  | *Total no. of food products undergoing product assessments at supermarkets* | *45,273* |  |  |  |  |  |  |  |  | 45,273.32 |
|  |  | Average time spent on product assessment per supermarket chain (hours) | 1,414.79 |  |  |  |  |  |  |  |  | 1,414.79 |
|  |  | Total costs of product assessment at supermarkets | 1,017,503.72 |  |  |  |  |  |  |  |  |  |
|  |  | ***Total costs of product assessment (start-up costs, 1st year)*** | ***1,017,503.72*** |  |  |  |  |  |  |  |  | 1,017,503.72 |
|  | **A.4** | **Costs of replaning store layout** | **Mean /Median / Adjustments (as reported)** | **Mean/Median (2024)** | **SE** | **MIN/LL** | **MAX/UL** | **alpha** | **Beta** | **Dist** | **Ersatz** | **Eroutput** |
|  |  | *Supermarket chains:* |  |  |  |  |  |  |  |  |  |  |
|  |  | Retail/store manager |  |  |  |  |  |  |  |  |  |  |
|  |  | *Adjusted by non-wage labour and overhead costs* |  | 71.15 |  |  |  |  |  |  |  |  |
|  |  | Number of retail/store manager per store | 1.00 |  |  |  |  |  |  |  |  |  |
|  |  | Time spent on replanning store layout at supermarkets (days) | 1.00 |  |  | 0.80 | 1.20 |  |  | Pert | 1.00 |  |
|  |  | Time spent on replanning store layout at supermarkets (hrs) | 7.60 |  |  |  |  |  |  |  |  |  |
|  |  | Stock control clerk |  |  |  |  |  |  |  |  |  |  |
|  |  | *Adjusted by non-wage labour and overhead costs* |  | 55.75 |  |  |  |  |  |  |  |  |
|  |  | Number of stock control clerk per store | 1.00 |  |  |  |  |  |  |  |  |  |
|  |  | Time spent on replanning store layout at supermarkets (days) | 1.00 |  |  | 0.80 | 1.20 |  |  | Pert | 1.00 |  |
|  |  | Time spent on replanning store layout at supermarkets (hrs) | 7.60 |  |  |  |  |  |  |  |  |  |
|  |  | Sales Assistant |  |  |  |  |  |  |  |  |  |  |
|  |  | *Adjusted by non-wage labour and overhead costs* |  | 27.76 |  |  |  |  |  |  |  |  |
|  |  | Number of sales assistants per store | 4.00 |  |  | 3.20 | 4.80 |  |  | Pert | 4.00 |  |
|  |  | Time spent on replanning store layout at supermarkets (days) | 1.50 |  |  | 1.20 | 1.80 |  |  | Pert | 1.50 |  |
|  |  | Time spent on replanning store layout at supermarkets (hrs) | 45.60 |  |  |  |  |  |  |  |  |  |
|  |  |  |  |  |  |  |  |  |  |  |  |  |
|  |  | Total time spent on replanning store layout at supermarkets (hrs) | 60.80 |  |  |  |  |  |  |  |  | 60.80 |
|  |  | Costs of replaning store layout at physical supermarkets | 9,155,207.45 |  |  |  |  |  |  |  |  |  |
|  |  | ***Total costs of replanning store layout*** | ***9,155,207.45*** |  |  |  |  |  |  |  |  | 9,155,207.45 |
|  | **A.5** | **Costs of on-going product assessment (after 1st year)** | **Mean /Median / Adjustments (as reported)** | **Mean/Median (2024)** | **SE** | **MIN/LL** | **MAX/UL** | **alpha** | **Beta** | **Dist** | **Ersatz** | **Eroutput** |
|  |  | General manager |  |  |  |  |  |  |  |  |  |  |
|  |  | *Adjusted by non-wage labour and overhead costs* |  | 179.80 |  |  |  |  |  |  |  |  |
|  |  | Number of staff per chain | 1.00 |  |  |  |  |  |  |  |  |  |
|  |  | Time spent on on-going product assessment per product | 0.13 |  |  | 0.08 | 0.17 |  |  | Pert | 0.13 |  |
|  |  | Total time spent on on-going product assessment (hours/chain/product) | 0.13 |  |  |  |  |  |  |  |  | 0.13 |
|  |  | *Supermarket chains:* | **Mean /Median / Adjustments (as reported)** | **Mean/Median (2024)** | **SE** | **MIN/LL** | **MAX/UL** | **alpha** | **Beta** | **Dist** | **Ersatz** | **Eroutput** |
|  |  | Number of new products |  |  |  |  |  |  |  |  |  |  |
|  |  | *Woolworths* | 1,500.00 |  |  | 1,200.00 | 1,800.00 |  |  | Pert | 1,500.00 |  |
|  |  | *Coles* | 1,421.00 |  |  | 1,136.80 | 1,705.20 |  |  | Pert | 1,421.00 |  |
|  |  | *Aldi* | 88.99 |  |  |  |  |  |  |  |  |  |
|  |  | *IGA* | 722.35 |  |  |  |  |  |  |  |  |  |
|  |  | Total number of new products at supermarket chains | 3,732.34 |  |  |  |  |  |  |  |  | 3,732.34 |
|  |  | **Calculating the proportion of new products compared to all products** | **Mean /Median / Adjustments (as reported)** | **Mean/Median (2024)** | **SE** | **MIN/LL** | **MAX/UL** | **alpha** | **Beta** | **Dist** | **Ersatz** | **Eroutput** |
|  |  | *Woolworths* | 7.50% |  |  |  |  |  |  |  |  |  |
|  |  | *Coles* | 5.68% |  |  |  |  |  |  |  |  |  |
|  |  | Average % of new products compared to all products | 6.59% |  |  |  |  |  |  |  |  | 6.59% |
|  |  | Number of new food products |  |  |  |  |  |  |  |  |  |  |
|  |  | *Woolworths* | 1,185.00 |  |  |  |  |  |  |  |  |  |
|  |  | *Coles* | 1,122.59 |  |  |  |  |  |  |  |  |  |
|  |  | *Aldi* | 70.30 |  |  |  |  |  |  |  |  |  |
|  |  | *IGA* | 570.66 |  |  |  |  |  |  |  |  |  |
|  |  | Total number of new food products at supermarket chains | 2,948.55 |  |  |  |  |  |  |  |  | 2,948.55 |
|  |  | Average time spent on ongoing product assessment per supermarket chain (hours) | 92.14 |  |  |  |  |  |  |  |  | 92.14 |
|  |  | ***Total costs of on-going product assessment*** | ***66,267.77*** |  |  |  |  |  |  |  |  | 66,267.77 |
|  | **A.6** | **Costs of handling complaints** | **Mean /Median / Adjustments (as reported)** | **Mean/Median (2024)** | **SE** | **MIN/LL** | **MAX/UL** | **alpha** | **Beta** | **Dist** | **Ersatz** | **Eroutput** |
|  |  | Penalties |  | 5,500 |  | 4,400.00 | 6,600.00 |  |  | Pert | 5,500.00 | 5500 |
|  |  | % of non-compliant stores in Y1 |  | 30% |  | 0.24 | 0.36 |  |  | Pert | 0.30 | 0.3 |
|  |  | % of non-compliant stores in Y2 |  | 20% |  | 0.16 | 0.24 |  |  | Pert | 0.20 | 0.2 |
|  |  | % of non-compliant stores in Y3 |  | 10% |  | 0.08 | 0.12 |  |  | Pert | 0.10 | 0.1 |
|  |  | Number of non-complaint store Y1 |  | 170 |  |  |  |  |  |  |  | 170 |
|  |  | Number of non-complaint store Y2 |  | 114 |  |  |  |  |  |  |  | 114 |
|  |  | Number of non-complaint store Y3 |  | 57 |  |  |  |  |  |  |  | 57 |
|  |  | Costs of handling complaints in Y1 |  | 936,966 |  |  |  |  |  |  |  | 936966 |
|  |  | Costs of handling complaints in Y2 |  | 624,644 |  |  |  |  |  |  |  | 624644 |
|  |  | Costs of handling complaints in Y3 |  | 312,322 |  |  |  |  |  |  |  | 312322 |
|  |  | Total costs of handling complaints |  | 1,873,933 |  |  |  |  |  |  |  | 1873933 |
| **3** | **Cost to Government** |  | **Mean /Median / Adjustments (as reported)** | **Mean/Median (2024)** | **SE** | **MIN/LL** | **MAX/UL** | **alpha** | **Beta** | **Dist** | **Ersatz** | **Eroutput** |
|  | **3.1** | **Cost of passing a legislation_1 (in 2010)** | **Mean /Median / Adjustments (as reported)** | **Mean/Median (2024)** | **SE** | **MIN/LL** | **MAX/UL** | **alpha** | **Beta** | **Dist** | **Ersatz** | **Eroutput** |
|  |  | Total costs of passing legislation | 1,090,000.00 | 1,557,630.48 | 110,744.67 |  |  | 197.83 | 7,873.74 | Gamma | 1,557,630.48 | 1,557,630.48 |
|  | **3.3** | **Cost of policy evaluation** | **Mean /Median / Adjustments (as reported)** | **Mean/Median (2024)** | **SE** | **MIN/LL** | **MAX/UL** | **alpha** | **Beta** | **Dist** | **Ersatz** | **Eroutput** |
|  |  | Total costs of policy evaluation | 500,000 |  |  | 400,000.000 | 600,000.000 |  |  |  | 500,000.000 |  |
|  | **3.2** | **Cost of monitoring** | **Mean /Median / Adjustments (as reported)** | **Mean/Median (2024)** | **SE** | **MIN/LL** | **MAX/UL** | **alpha** | **Beta** | **Dist** | **Ersatz** | **Eroutput** |
|  |  | Government officer |  |  |  |  |  |  |  |  |  |  |
|  |  | *Adjusted by non-wage labour and overhead costs* |  | 94.86 |  |  |  |  |  |  |  |  |
|  |  | Number of staff | 1.00 |  |  |  |  |  |  |  |  |  |
|  |  | Staff cost per year | 187,446.168 |  |  |  |  |  |  |  |  |  |
|  |  | Time spent on policy monitoring (FTE per store visit/year) | 0.007 |  |  | 0.006 | 0.008 |  |  | Pert | 0.007 | 0.01 |
|  |  | Staff cost per year per store checked | 1,312 |  |  |  |  |  |  |  |  | 1,312.12 |
|  |  |  |  |  |  |  |  |  |  |  |  |  |
|  |  | Proportion of stores checked | 7.00% |  |  | 5.00% | 50.00% |  |  | Pert | 0.138 | 0.14 |
|  |  | Number of stores checked | 568 |  |  |  |  |  |  |  |  | 567.86 |
|  |  | ***Total costs of monitoring*** | ***745,100.08*** |  |  |  |  |  |  |  |  | 745,100.08 |
|  | **3.3** | **Cost of targeted mass media campaign** | **Mean /Median / Adjustments (as reported)** | **Mean/Median (2024)** | **SE** | **MIN/LL** | **MAX/UL** | **alpha** | **Beta** | **Dist** | **Ersatz** | **Eroutput** |
|  |  | Tobacco Plain Packaging Advertising Campaign targeted towards the business sector ($2012-13) |  | 954,482 |  | 763,585.657 | 1,145,378.486 |  |  | Pert | 954,482.072 | 954,482.07 |
